# Pro- and anti-inflammatory cytokines: the hidden keys to autoimmune gastritis therapy

**DOI:** 10.3389/fphar.2024.1450558

**Published:** 2024-08-13

**Authors:** Greta Cascetta, Giorgia Colombo, Gianmarco Eremita, Joe G. N. Garcia, Marco Vincenzo Lenti, Antonio Di Sabatino, Cristina Travelli

**Affiliations:** ^1^ Department of Pharmaceutical Sciences, University of Pavia, Pavia, Italy; ^2^ Division of Hematology/Oncology, Department of Medicine, Weill Cornell Medicine, Cornell University, New York, NY, United States; ^3^ Center for Inflammation Science and Systems Medicine, University of Florida Scripps Research Institute, Jupiter, FL, United States; ^4^ First Department of Internal Medicine, IRCCS San Matteo Hospital Foundation, University of Pavia, Pavia, Italy

**Keywords:** autoimmune atrophic gastritis, cytokine, inflammation, immune system, therapy

## Abstract

Autoimmune gastritis (AIG) is an autoimmune disorder characterized by the destruction of gastric parietal cells and atrophy of the oxyntic mucosa which induces intrinsic factor deficiency and hypo-achlorhydria. AIG predominantly affects the antral mucosa with AIG patients experiencing increased inflammation and a predisposition toward the development of gastric adenocarcinoma and type I neuroendocrine tumors. The exact pathogenesis of this autoimmune disorder is incompletely understood although dysregulated immunological mechanisms appear to major contributors. This review of autoimmune gastritis, an unmet medical need, summarizes current knowledge on pro- and anti-inflammatory cytokines and strategies for the discovery of novel biomarkers and potential pharmacological targets.

## 1 Introduction

Autoimmune gastritis (AIG) is an autoimmune disorder characterized by the destruction of gastric parietal cells, leading to the atrophy of the oxyntic mucosa. This process results in intrinsic factor deficiency and hypo-achlorhydria, thereby causing malabsorption of iron, vitamin B12, and other micronutrients (Rugge et al., 2002; Lenti et al., 2020). In AIG, the gastric proton pump, H^+^/K^+^ ATPase, is the major autoantigen recognized by autoreactive T cells which, by stimulating B cells, stimulates the production of anti-parietal cell antibodies, the serological hallmark of AIG. Unlike chronic gastritis related to *Helicobacter Pylori* (HP) infection that primarily affects the antral mucosa, autoimmune gastritis tends to spare the antral mucosa predominantly affecting the gastric body (Sugano et al., 2015). The relationship between HP infection and the pathogenesis of AIG remains unclear. HP infection may exacerbate AIG, as the destruction of parietal cells caused by HP-related inflammation could expose molecular patterns of the H^+^/K^+^ ATPase pump. A case report demonstrated that HP-related gastritis ultimately transitioned into AIG and that the progression of AIG led to the spontaneous disappearance of the HP infection due to worsening gastric atrophy (Kotera et al., 2023). Conversely, murine AIG was promoted by a CD4^+^ Th-1 response, which appears to be downregulated in mice infected with HP due to Th-2 immune responses and transforming growth factor β (Ohana et al., 2003). This is consistent with a recent case report in a woman with HP-related gastritis who developed AIG after eradication therapy, with a rapid progression of the atrophic pattern in the corpus within 3 years (Ihara et al., 2023). These data suggest that HP gastritis may have a suppressive effect on AIG pathogenesis. Supporting this hypothesis, the prevalence of AIG is very low in Asian countries, where the prevalence of HP infection is high (Quach et al., 2018).

Although the pathogenesis of AIG remains unclear, chronic inflammation of the gastric mucosa is likely involved and is related to the presence of anti-parietal cell autoantibodies present in 60%–70% of AIG patients (Osmola et al., 2023). These autoantibodies, specifically those against H^+^/K^+^ ATPase and intrinsic factor, demonstrate complement-dependent cytotoxic activity against gastric parietal cells in *in vitro* (De Aizpurua et al., 1983). This mechanism, however, fails to address the portion of AIG patients that are parietal cell antibodies (PCA)-negative (Lenti et al., 2017; Lenti et al., 2018) where cell-mediated immunity likely contributes to the development of AIG. AIG pathogenesis may be related to the exposure of molecular patterns by autoreactive T-cells leading to PCA antibody production (Katakai et al., 1998). More recently, vitamin D deficiency has been proposed to be related to AIG pathogenesis, since the vitamin D receptor regulates T-cell activation and maturation (Fernandez Lahore et al., 2020). Although, the pathogenesis of AIG is not entirely defined, a CD4^+^ Th-1 response and the autoreactivity of Th-1 cells have been postulated. In any case, the increase in inflammation in the corpus leads to the destruction and subsequent loss of parietal cells. Notably, Mario D’Elios et al. firstly demonstrated in humans that the T cell-dependent activation of B cells stimulates the production of anti-parietal cell antibodies, the serological hallmark of AIG. Indeed, the 25% of the CD4^+^ from the gastric corpus of AIG patients proliferated in response to H^+^, K^+−^ATPase and shows a Th1 profile, producing TNFα and provides help for B-cell immunoglobulin production, suggesting that the activation of the proton pump–specific Th1 cytotoxic T cells in the gastric mucosa can represent an effector mechanism for the target cell destruction in AIG (D’Elios et al., 2001). These data suggest that the Th1-dependent activation of human gastric cytotoxic mechanisms with IFN and TNFα production are crucial in the inflammatory damaging mechanisms of human autoimmune gastritis (D’Elios et al., 2001).

Moreover, genetic predisposition has been described, since family history of AIG has been reported in patients (Lenti et al., 2019). The heightened state of inflammation in AIG patients predisposes them to the development of gastric adenocarcinoma and type I neuroendocrine tumors (Lahner et al., 2019; Lenti et al., 2019) highlighting the need for early diagnosis and discovery of novel biomarkers or therapeutics. This review aims to recapitulate the role of the major cytokines that are dysregulated in AIG, shedding the light on the contribution of each cytokine to the disease, with the aim to finally highlight potential novel therapeutics for AIG.

## 2 Cytokine deregulation in AIG

**FIGURE 1:**
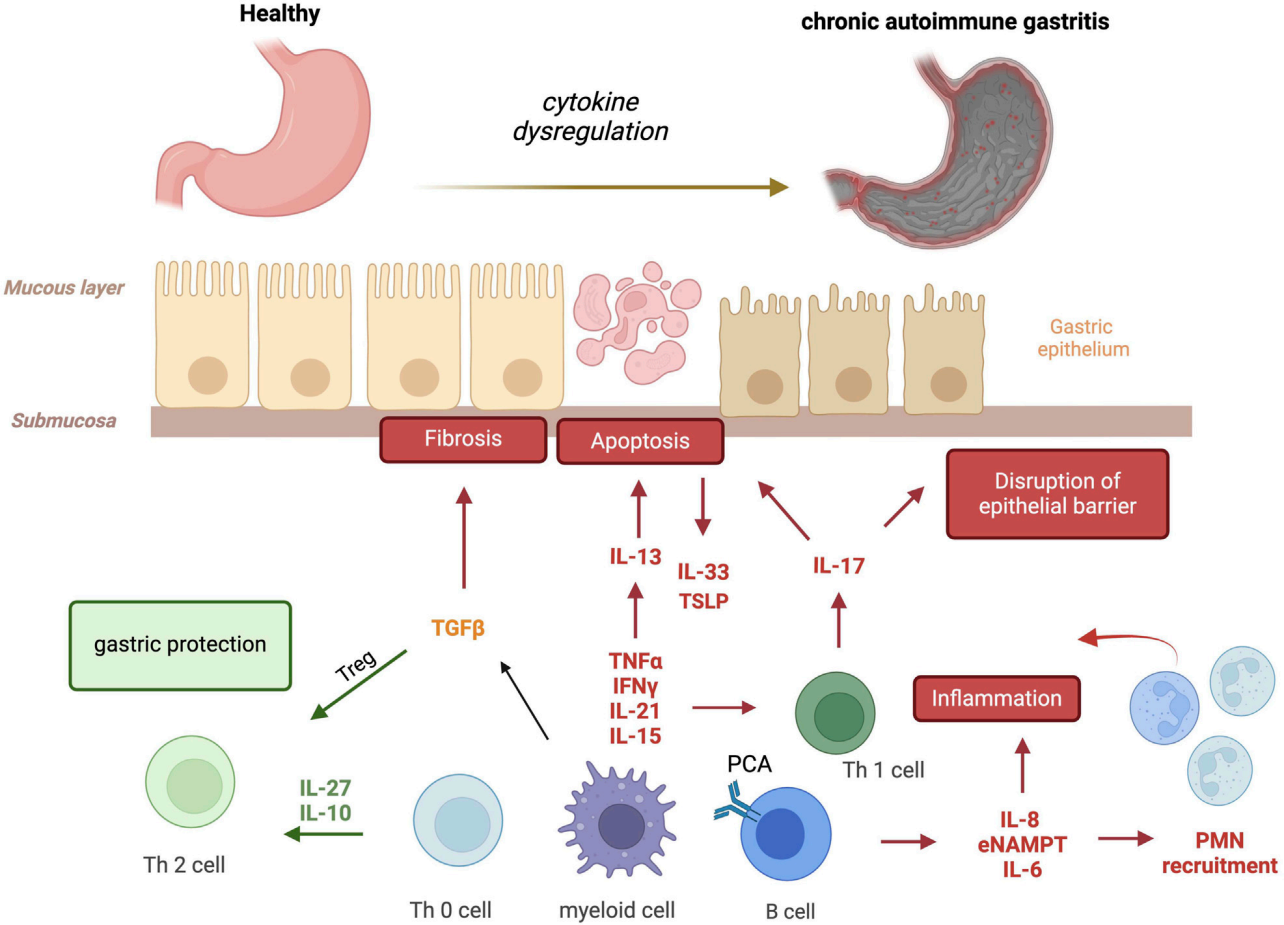
Cytokines involved in autoimmune gastritis. Several pro- and anti-inflammatory cytokines are produced by activated autoreactive T cells, macrophages and B cells, amplifying the immune response and favoring parietal cell apoptosis, the disruption of epithelial barrier and fibrosis. In red (pejorative cytokines), in green (protective cytokines) and in yellow (real contribution to be define) (IFNγ, interferon-gamma; TNF-α, Tumor Necrosis Factor-α; IL-21, Interleukin-21; IL-15, Interleukin-15; TGFβ, Transforming Growth Factor-β; IL-27, Interleukin-27; IL-10, Interleukin-10; IL-13, Interleukin-13; IL-33, Interleukin-33; TSLP, Thymic Stromal Lymphopoietin; IL-17, Interleukin-17; IL-8, Interleukin-8; eNAMPT, extracellular nicotinamide phosphoribosyl transferase; IL-6, Interleukin-6; PMN, polymorphonuclear neutrophils).

The immune system is the first line of defense in our body, composed of immune cells that protect from infections. These immune cells are part of a complex network involving both innate and adaptive immunity. Innate immunity is an antigen-independent, non-specific defense mechanism that the host uses immediately upon exposure to pathogens. In contrast, adaptive immunity is antigen-dependent and antigen-specific, requiring a latency period for develop of the immune response following exposure to antigen. Innate and adaptive immunity are not mutually exclusive mechanisms of host defense; but necessary and complementary necessary for responding to disease and infection. However, immune system malfunction with targeting of healthy cells and organs, results in autoimmune disease. This occurs in each organ system including the gastrointestinal tract (Martinelli et al., 1996; Burns et al., 2019) producing disorders such as autoimmune gastritis. This review will discuss the current understanding of the role of pro- and anti-inflammatory cytokines in controlling the pathogenesis and/or progression of AIG (Figure 1).

### 2.1 Interleukin-6 (IL-6)

IL-6 is a cytokine with pleiotropic effects on immune response and hematopoiesis. The levels of IL-6 increase after infections and tissue damage, playing a crucial role in controlling the acute phase of inflammatory responses. IL-6 dysregulation is observed in several chronic inflammatory conditions with tocilizumab, a humanized anti-IL-6 receptor antibody, approved for the treatment of rheumatoid arthritis and juvenile idiopathic arthritis (Burmester et al., 2017; He et al., 2024). IL-6 dysregulation is also observed in gastrointestinal tract disorders (Garbers and Lokau, 2024), although the role of IL-6 in autoimmune gastritis remains unclear. *Helicobacter pylori* enhances IL-6 production, creating a positive feedback loop between gastric epithelial cells and macrophages, ultimately predisposing to gastric carcinogenesis (Yu et al., 2024). Conversely, Yang et al. found a statistically significant variation in cytokine expression between HP-positive and HP-negative gastritis. Specifically, they observed lower expression levels of IL-6 in HP-positive patients compared to those with HP-negative gastritis (Yang et al., 2024). These findings suggest that while HP infection can induce IL-6 production, IL-6 may be more involved in HP-negative gastritis, indicating a potential role for IL-6 in autoimmune disorders such as autoimmune atrophic gastritis. Supporting this, IL-6 has been detected in the supernatants of AIG corpus mucosa, with *in vitro* modulation of also observed (Lenti et al., 2022). Thus, although the exact IL-6 contribution to AIG is incompletely defined, tocilizumab could represent a potential novel AIG therapeutic option, even though safety issues have been raised (Ozaka et al., 2023).

### 2.2 Interferon-γ (IFNγ)

IFNγ is a classical proinflammatory mediator secreted by both lymphoid and myeloid cells, primarily by CD4^+^ and CD8^+^ T cells (Tau and Rothman, 1999). In the context of AIG, H^+^/K^+^ ATPase-specific CD4^+^ T cells are involved, whereas CD8^+^ T cells do not appear to be affected (Suri-Payer and Cantor, 2001). In a murine model of AIG, increased levels of IFNγ^+^ CD4^+^ T cells have been observed (Suri-Payer and Cantor, 2001), a finding validated in the TxA23 mouse model, where IFNγ is directly involved in the death of gastric epithelial cells, leading to the development of chronic atrophic gastritis (Tu et al., 2012). These data suggests that the production of high levels of IFNγ in AIG may regulate the recruitment and infiltration of T cells in the stomach and inhibit Treg function. Notably, IFNγ promotes parietal cell atrophy with metaplasia during the progression of gastritis. Gastric epithelial cells expressing the IFNγ receptor in the basolateral membrane and gastroids died when treated with IFNγ whereas mice lacking IFNγ (IFNγ^−/−^ mice) exhibited abrogation of atrophy and metaplasia. and *In vivo* administration of IFNγ inhibitors was protective against gastritis induction (Osaki et al., 2019). Collectively, these findings highlight IFNγ as a key mediator in the severity of AIG.

### 2.3 Interleukin-27 (IL-27)

Interleukin-27, a member of the IL-12 family involved in controlling Th1 responses, is a controversial cytokine as it also possesses anti-inflammatory properties by directly modifying the effector functions of CD4^+^ and CD8^+^ T cells (Schneider et al., 2011). This cytokine is actively secreted by monocytes, macrophages, dendritic cells, and T cells (Povroznik and Robinson, 2020.). Notably, IL-27 acts as a growth and survival factor for T-cells, positively influencing many aspects of their function (Kim et al., 2013). Specifically, IL-27 regulates IL-10 production, IFNγ and IL-17A, which directly act on the gastric epithelium and promote parietal cell atrophy, indicating that IL-27 may play a key role in controlling the production of other cytokines dysregulated in AIG (Bockerstett et al., 2020). Notably, in humans, IL-27 expression levels are high following HP^+^ infection, however, these findings have not been fully replicated in the TxA23 mouse model of AIG, which expresses a T-cell receptor against the H^+^/K^+^ adenosine triphosphatase α chain and develops autoimmune gastritis. In this model, researchers administered high-dose tamoxifen to induce parietal cell atrophy and spasmolytic polypeptide-expressing metaplasia (SPEM) and found that IL-27-deficient mice developed more severe gastritis, atrophy and SPEM compared to control mice. Moreover, the administration of recombinant IL-27 significantly reduced the severity of inflammation and SPEM in mice with gastritis (Bockerstett et al., 2018). This effect was mediated by IL-27 acting exclusively on stomach-infiltrating CD4^+^ T cells to suppress the expression of inflammatory genes (Bockerstett et al., 2020). In conclusion, IL-27 is a cytokine with both pro- and anti-inflammatory properties and the data obtained so far suggest a possible role for IL-27 in the pathogenesis of HP^+^ gastritis, although its role in AIG remains to be fully elucidated.

### 2.4 Interleukin-13 (IL-13)

IL-13, a pro-inflammatory cytokine intricately involved in allergic responses, is primarily synthesized by lymphoid cells, macrophages and mast cells (de Vries, 1998). In the context of AIG, several investigations have studied the role of IL-13 in the development of spasmolytic polypeptide-expressing metaplasia (SPEM) during AIG in response to infection (Noto et al., 2022). The clinical evidence underscores significantly elevated IL-13 levels in the blood of AIG patients compared to healthy individuals. Similarly, IL-13 expression is increased in the gastric mucosa of TxA23 mice with AIG with a correlation with the level of metaplasia with mast cells identified as the predominant source of IL-13 *in vivo*. IL-13 directly influences gastric epithelial cells, augmenting organoid size and enhancing epithelial cell viability (Noto et al., 2022). Mice with AIG lacking the IL-13 receptor fail to exhibit neck cell expansion or metaplasia and anti-mouse IL-13-neutralizing antibodies inhibit and reverse disease progression during chronic gastritis (Noto et al., 2022). Collectively, these findings suggest that IL-13 is generated by diverse subsets of immune cells during chronic gastritis, promoting changes in gastric epithelial cells associated with gastric cancer. *In vivo*, IL-13-neutralizing antibodies reduce the severity of metaplasia during AIG. Further exploration of IL-13 appears to be warranted, particularly given the development of neutralizing antibodies such as tralokinumab, lebrikizumab, cendakimab, and eblasakimab (Lytvyn and Gooderham, 2023).

### 2.5 Interleukin-17 (IL-17)

IL-17 is an inflammatory cytokine secreted by a distinct subset of CD4^+^ T helper cells known as Th-17 cells. CD4^+^ T cells can differentiate into various T helper cell subsets, each with unique cytokine profiles and effector functions. Traditionally, T cells were classified as Th-1 or Th-2 based on the cytokines they produce, however, a third Th-17 producing subset (Korn et al., 2009) has been identified that plays a crucial role in eliminating bacteria and fungi by inducing defensin production. The IL-23/IL-17 axis is implicated in chronic inflammation related to the pathogenesis of inflammatory and autoimmune disorders, including chronic autoimmune diseases, kidney inflammation, and intestinal inflammation (Kuwabara et al., 2017). In the context of AIG, IL-17A and IL-17F are produced in the corpus of AIG patients following activation with H^+^/K^+^-ATPase and that serum IL-17A, IL-17F and IL-17E levels are significantly elevated in AIG patients (Della Bella et al., 2022). IL-17 secretion, mediated by self-immune responses of CD4^+^ T cells, targets the H^+^/K^+^ ATPase and activates the NLRP3 inflammasome pathway (Krohn et al., 2018; Zhang et al., 2023) leading to parietal cell death and contributing to AIG development.

Studies using mouse models of autoimmune-mediated atrophic gastritis, such as the TxA23 model, have established a correlation between the severity of gastric atrophy and the pro-inflammatory cytokine IL-17. Both IL-17 and IFNγ exacerbate AIG progression, although the absence of IL-17 or IFNγ does not completely prevent its occurrence (Bockerstett et al., 2018). Elevated IL-17A levels are often observed in patients with AIG or gastric cancer and correlate with disease severity in mice with chronic atrophic gastritis, specifically causing caspase-dependent gastric organoid degeneration. Notably, an IL-17A neutralizing antibody reduced parietal cell atrophy and metaplasia in a preclinical AIG model (Bockerstett et al., 2018) suggesting that IL-17A induces caspase-dependent apoptosis of parietal cells *in vivo*, highlighting the potential use of anti-IL-17 therapy in AIG.

### 2.6 Interleukin-21 (IL-21)

IL-21 is a type-I cytokine produced by T-cells, exerting potent effects on various immune cells, including NK, T, B, and NKT cells. IL-21 influences both lymphoid and myeloid populations and can either positively or negatively regulate immune responses depending on the specific context (Coquet et al., 2007). Few studies have explored the role of IL-21 in t AIG progression, however, in a mouse model of rapidly progressing AIG produced by neonatal thymectomy on programmed cell death 1-deficient (NTx-PD-1^−/−^) mice on the BALB/c background, expression of IFNγ, TNFα, and IL-21 were elevated in the inflamed gastric mucosa (Nishiura et al., 2013). Additionally, *in vivo* administration of an anti-IL-21 antibody suppressed disease exacerbation in these mice (Nishiura et al., 2013). These initial findings suggest that IL-21 may play a critical role in the advancement of autoimmune gastritis but requires studies to determine whether IL-21 overexpression is a primary promoter of AIG.

### 2.7 Interleukin-33 (IL-33)

IL-33 is a cytokine belonging to the IL-1 family, acting as an alarmin in response to cellular damage or stress. IL-33 is a nuclear protein (Baekkevold et al., 2003) that is also released into the extracellular space through several mechanisms, primarily associated with cellular damage or stress (Cayrol and Girard, 2022), thus acting as a dual-function molecule. IL-33 is expressed in cells that are in contact with the environment, such as endothelial and epithelial cells (Moussion et al., 2008), and acts as an early inducer of inflammation by binding to the cell-surface receptor ST2 (suppression of tumorigenicity 2), leading to the activation of intracellular signaling pathways similar to those used by IL-1 (Schmitz et al., 2005). This binding promotes the production of Th2-associated cytokines, contributing to the regulation of immune responses, inflammation, and tissue repair. IL-33 has been implicated in the pathogenesis of several diseases where it plays a dual protective/pathogenic role (Seo et al., 2017). For example, IL-33 contributes to host defense against parasitic and bacterial infections and exerts cardioprotective effects (Palmer and Gabay, 2011; Erfurt et al., 2021), however, in chronic gastritis, IL-33 release after parietal cell loss induces IL-13 production from ILC2s to promote intestinal goblet cell differentiation in mice (Privitera et al., 2024). Targeting cytokines, such as IL-33, was effective in modulating the downstream recruitment and activation of immune cells, such as eosinophils, M2 macrophages, and ILC2, reducing gastritis and downstream gastric metaplasia.

### 2.8 Interleukin-8 (IL-8)

IL-8, a member of the CXC chemokine family, is a chemoattractant for neutrophils and lymphocytes, and controls cell proliferation and migration. IL-8, stimulated by *H. pylori* infection, induces the recruitment of neutrophils, which secrete proinflammatory cytokines such as TNF-α, IFNγ, and IL-1β. The cytokine response in gastric mucosa is thought to be Th1-predominant, characterized by the accumulation of IFNγ, which controls IL-8 expression (Bosco et al., 1994). The secretion of IL-8 by epithelial cells is a key factor in host defenses and mucosal IL-8 production in HP infection may be an important factor in the immunopathogenesis of peptic ulcer disease and also be of relevance to gastric carcinogenesis (Crabtree and Lindley, 1994). IL-8 expression levels are lower in HP-positive gastritis patients compared to HP-negative, suggesting IL-8 may be increased in AIG patients.

### 2.9 Interleukin-15 (IL-15)

IL-15 is a cytokine belonging to the IL-2 family that plays a critical role in the development, survival and activation of various immune cells, particularly natural killer cells and memory CD8^+^ T cells (Waldmann, 2006). IL-15 is produced by a variety of cells, including monocytes, macrophages, dendritic cells and fibroblasts (Budagian et al., 2006), and is essential for the homeostasis and function of both the innate and adaptive immune systems. IL-15 binds to the IL-2 receptor β and γ domains and shares many biological activities with IL-2, but also has a specific unique chain called IL-15 receptor α (Giri et al., 1995). Due to its role in immune cell activation and proliferation, dysregulation of IL-15 can contribute to the pathogenesis of several diseases such as cancer, infectious diseases, and inflammatory and autoimmune diseases. Activation of NK and CD8^+^ cells induced by IL-15-mediated recruitment is responsible of promoting antitumor immunity (Kurz et al., 2022) and IL-15-induced T-cell activation promotes the production of pro-inflammatory cytokines such as IFNγ and TNF in inflammatory bowel diseases (Liu et al., 2000). Furthermore, *ex vivo* levels of IL-15 were found to be increased in the gastric mucosa of AIG patients after 24 h of culture compared to healthy controls (Santos Savio et al., 2015). Notably, the anti-inflammatory zinc-1-carnosine treatment for 24 h significantly reduced IL-15 production, aiding in the restoration of gastric healing (Lenti et al., 2022). To date, no other data are available regarding the role of IL-15 in gastric disorders.

### 2.10 Interleukin-10 (IL-10)

IL-10 is a key anti-inflammatory mediator that ensures protection of the host from pathogens, while also playing roles in wound healing and autoimmunity. IL-10 is produced by CD4^+^ T cells, CD8^+^ T cells, monocytes, and B cells after its secretion IL-10 is a regulator of the immune response. In detail, IL-10 inhibits the expression of many pro-inflammatory cytokines, chemokines, and chemokine receptors, and mediates allergen tolerance. In myeloid cells, IL-10 has anti-inflammatory properties on macrophages and T cells (Ip et al., 2017). Notably, of great importance for AIG, IL-10 can inhibit the synthesis of pro-inflammatory cytokines such as IFNγ (Torisu et al., 2008). In the AIG murine model, dendritic cells induce regulatory T cells and IL-10, thereby participating in the protection against AIG (Suri-Payer and Cantor, 2001; Torisu et al., 2008). IL-10 has also been correlated with HP^+^ gastritis, where immunosuppressive CD19^+^IL-10^+^ B cells were found to be significantly higher in HP-positive patients compared to uninfected patients (Nahid-Samiei et al., 2020). The limited data on IL-10 in the context of AIG suggest a protective role for IL-10.

### 2.11 Tumor necrosis factor-α (TNFα)

TNFα is a pro-inflammatory cytokine, member of the TNFα superfamily, secreted by activated macrophages/monocytes, as well as by lymphocytes, Kupffer cells, peritoneal macrophages, and vascular smooth muscle (Parameswaran and Patial, 2010). TNFα plays a crucial role in the regulation of immune cells, inflammation, necrosis and apoptosis (Jang et al., 2021) and is rapidly produced in response to infections or other stressors, LPS and viral particles (acute phase reactant) (Peng et al., 2022). TNFα upregulation is implicated in various pathologies involving inflammation and immune system dysregulation, including rheumatoid arthritis, cancer and type 2 diabetes (Parameswaran and Patial, 2010). Recently, TNFα involvement in AIG was suggested by significantly increased TNFα levels in organ culture supernatants from a cohort of 24 patients with AIG compared to healthy controls (Lenti et al., 2022) which was reduced by 24 h treatment with short Thymic stromal lymphopoietin (TSLP) and zinc-I- carnosine. Therefore, TNFα may support the inflammatory process leading to gastric atrophy, while TSLP lamina propria overexpression may work as modest compensatory mechanism.

### 2.12 Extracellular nicotinamide phosphoribosyltrasferase (eNAMPT)

eNAMPT is a novel cytokine and damage-associated molecular pattern protein (DAMP) that is also known as pre-B-cell colony-enhancing factor or visfatin (Sommer et al., 2008), NAMPT is an essential enzyme for mammalian metabolism since is the enzyme controlling the rate limiting step of NAD salvage synthesis pathway. When NAMPT is secreted in the extracellular space (eNAMPT) it plays an important role in the regulation of insulin secretion in pancreatic β-cells and as an immunomodulatory cytokine involved in the regulation of inflammatory responses (Moschen et al., 2010; Managò et al., 2019; Colombo et al., 2022). Indeed, eNAMPT serves as a DAMP by promoting the production and release of other pro-inflammatory cytokines such as TNFα, IL-6, and IL-1β following eNAMPT binding to Toll-like receptor 4 (TLR4) on immune cells, leading to the activation of NF-κB and other transcription factors that upregulate the expression of pro-inflammatory cytokines (Camp et al., 2015; Garcia et al., 2022). Elevated levels of eNAMPT are associated with chronic inflammatory diseases such as rheumatoid arthritis, systemic lupus erythematosus, inflammatory bowel disease, breast cancer, acute respiratory distress syndrome (ARDS) and obesity-related inflammation including NASH (Sun et al., 2014; 2023; Colombo et al., 2020; Travelli et al., 2023; Tumurkhuu et al., 2023). Moreover, NAMPT over-expression contributes to the persistence of inflammation by sustaining the production of inflammatory cytokines (He et al., 2021). In AIG, the role of eNAMPT has been investigated only recently, where NAMPT transcript levels were significantly increased in AIG gastric corpus biopsies compared with healthy controls, and particularly eNAMPT, in the supernatants of organ culture biopsies was significantly increased in AIG. Moreover, it was also demonstrated that NAMPT exerts a pro-inflammatory effect on normal gastric mucosa by increasing IFNγ and IL-6 mucosal transcript (Lenti et al., 2022). Although further studies are necessary to clarify the role of eNAMPT in AIG pathogenesis, the availability of an eNAMPT-neutralizing mAb (ALT-100), currently in Phase 2A clinical trials for ARDS (NCT05938036), heightens the feasibility of an anti-eNAMPT therapeutic strategy in AIG.

### 2.13 Transforming growth factor-beta (TGFβ)

TGFβ is a key factor in controlling immune homeostasis and the fibrotic process (Biernacka et al., 2011). Many different cell types, including macrophages, lymphocytes, endothelial cells, epithelial cells, and fibroblasts secrete TGFβ isoforms at sites of inflammation. The specific cellular origins of TGFβ are dependent on the type of injury and the cellular composition of the affected organ. Due to its central role in regulating homeostasis in the stomach, TGFβ could be an important regulator of gastric diseases. Notably, TGFβ is extensively implicated in the regulation of fibrosis, indeed, fibroblasts are major targets of TGFβ and the increased amount of TGFβ in the stomach can lead to increase of the expression of matrix metalloproteinases (MMPs) which are specialized extracellular matrix component proteins that may serve to initiate signaling responses promoting fibrosis. In the context of inflammation, TGFβ controls regulatory T-cells, especially the Treg subtype, thus contributing to the regulation of continuous inflammation (Dituri et al., 2021). In the field of gastritis, the adoptive transfer of Tregs reduced the development of autoimmune gastritis in a mouse model. The authors demonstrated that TGFβ-expressing Tregs were effective in suppressing inflammation and decreasing the severity of the disease (Nguyen et al., 2014). This data in mice demonstrate that Tregs were effective at suppressing disease progression during the late stages of AIG. Accordingly, naïve T-cells specific for the autoantigen H^+^/K^+^ ATPase can be converted to Foxp3^+^ T regulatory cells when stimulated in the presence of TGFβ. This TGFβ-induced Tregs are effective at preventing specific autoimmunity in a murine model of AIG (Stummvoll et al., 2008). While TGFβ targeting strategies may be beneficial AIG, the real contribution of this fibrotic factor in the corpus of patients remain to be clarified.

### 2.14 Thymic Stromal Lymphopoietin (TSLP)

Thymic stromal lymphopoietin (TSLP) is an epithelial-derived factor which signal is processed via a TSLP receptor, a heterodimer of the IL-7 receptor α chain and the TSLPR chain. The TSLPR chain is closely related to the common receptor γ chain that is expressed on a wide range of cell types in the adaptive and innate immune system (He and Geha, 2010), but also in intestinal and epithelial cells, stromal cells and mast cells (He and Geha, 2010). TSLP is an important cytokine in IL-2 family, which stimulates thymocytes and promotes B cell lymphopoiesis. In immune cells, TSLP is mainly expressed by T cells, dendritic cells and B cells where TLSP acts as master regulator of Th-2-type inflammation immune responses and Th-17-mediated autoimmune responses (Nakajima et al., 2020). Importantly, depending on the context and tissue in which it is expressed, TSLP exhibits dual effects including promoting inflammation aggravation or maintaining homeostasis. In support of this, in the gastric apparatus TLSP has found to be correlated as both suppressor and promoter of tumor growth (He and Geha, 2010). In the AIG murine model, defects of TSLPR produce anti-parietal cell antibodies, gastric mucosal inflammation and AIG worsens. In AIG affected mice, TSLP negatively regulates the production of IL-12/23p40 to promote Th1-type autoimmunity in the corpus. Notably, this finding has been recapitulated also in humans, since TSLP is more expressed in AIG biopsies compared with HC and HP^+^ gastritis (Lenti et al., 2022).

## 3 Possible novel therapeutics for the treatment of AIG

**FIGURE 2:**
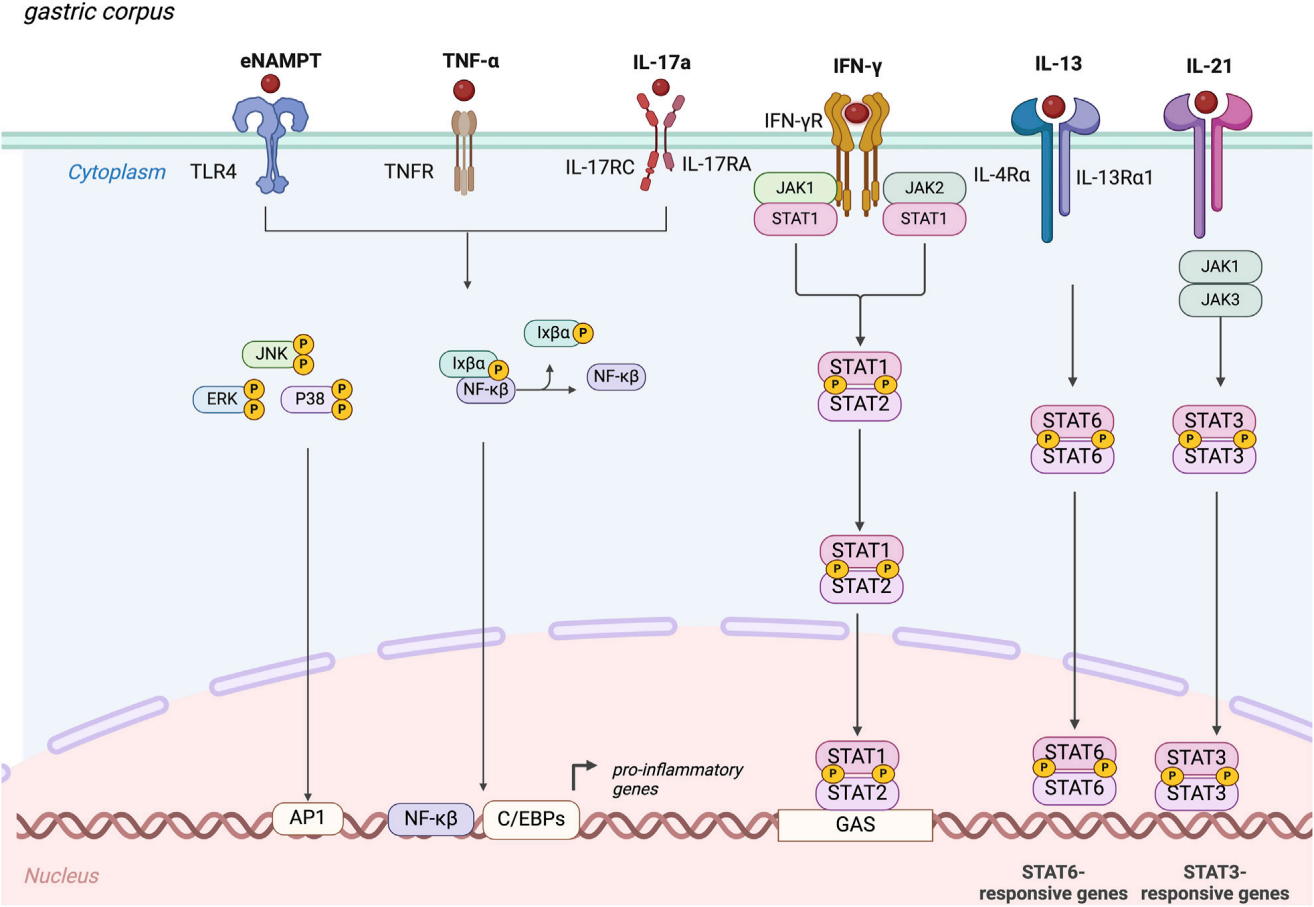
Molecular mechanisms elicit by the main pejorative cytokines involved in autoimmune gastritis. IFNγ, interferon-gamma; TNF-α, Tumor Necrosis Factor-α; IL-21, Interleukin-21; IL-13, Interleukin-13; IL-17A, Interleukin-17A; eNAMPT, extracellular nicotinamide phosphoribosyl transferase, JAK, Janus kinase; JNK, c-Jun N-terminal kinases; ERK, Extracellular signal-regulated kinases; P38, mitogen-activated protein kinases; NF-κB, nuclear factor kappa-light-chain-enhancer of activated B cells; STAT, Signal transducer and activator of transcription.

Despite an autoimmune pathogenesis, there are no currently approved biological therapies for AIG, a major unmet need, and AIG treatment mainly involves micronutrient supplementation. Additional research is needed to better understand the role of pro-inflammatory cytokines as potential therapeutic targets for AIG. Here, we clarify the possible pejorative cytokines controlling worsening of the disease (Table 1), and suggest that IL-6, IFNγ, TNFα, IL-13, IL-17, and eNAMPT could be promising targets for future treatments. These cytokines act through the binding of specific membrane receptors and share in part their molecular pathways that are summarized in Figure 2.

**TABLE 1:** Summary of the current knowledge on cytokine roles, levels and possible future medications in AIG.

Cytokine/factor	Main findings in Autoimmune gastritis	Levels in AIG	Role in AIG	Putative future biological therapy	References
IL-6	Increase in HP + gastritis and in autoimmune gastritis, detected in corpus of AIG patients.	Increased	To be define	Tocilizumab	Lenti et al. (2022), Yu et al. (2024)
IFNγ	Increased levels of IFNγ^+^ CD4^+^ T cells in AIG mice; IFNγ is directly involved in the death of gastric epithelial cells, IFNγ inhibitor are protective in murine models.	Increased	Pejorative	Emapalumab	Suri-Payer and Cantor (2001), Tu et al. (2012)
IL-27	IL-27 expression levels are high following HP infection, in IL-27-deficient mice developed more severe gastritis, IL-27 seems to be protective in mice.	Not affected	To be define	—	Bockerstett et al., 2018 (2020)
IL-10	In AIG mice, dendritic cells have been shown to induce regulatory T cells and IL-10 *in vitro*, thereby participating in the protection against AIG.	Not affected	Protective (to be verify)	—	Wang et al. (2019)
IL-13	IL-13 expression has been observed increased in the gastric mucosa of TxA23 mice with AIG with a correlation with the level of metaplasia; neutralization of IL-13 using anti-mouse IL13 antibodies inhibits and reverses disease progression during chronic gastritis.	Increased	Pejorative	Tralokinumab, lebrikizumab, cendakimab, and eblasakimab	Noto et al. (2022)
IL-17	expression IL-17A increased in patients with AIG; actively secretion of IL-17A n the gastric mucosa of AIG	Increased	Pejorative	Secukinumab, ixekizumab, and brodalumab	Krohn et al. (2018), Zhang et al. (2023)
IL-21	In mice, *in vivo* administration of anti-IL-21 antibodies, there was a discernible suppression of disease exacerbation in these mice	To be define	Pejorative	NNC01140006 (anti-Il-21 antibody) (Not approved; phase II)	Safety and Tolerability of NNC01140006, an Anti-IL-21 Monoclonal Antibody, at Multiple s.c. Dose Levels in Patients with Rheumatoid Arthritis (2024)
IL-33	IL-33 is released after parietal cell death; reduction of IL-33 reduced gastritis and downstream gastric metaplasia in mice.	Increased	Pejorative (To be validate)	Tozorakimab	England et al. (2023)
IL-8	IL-8 stimulated by *H. pylori* infection	To be define	To be define	—	Crabtree and Lindley (1994)
IL-15	Levels of IL-15 are increased in the gastric mucosa of AIG patients	Increased	To be define	—	Santos Savio et al. (2015); Lenti et al. (2022)
TNFα	TNFα was significantly higher in AIG patients	Increased	Pejorative	Infliximab, adalimumab, etanercept, golimumab, certolizumab	Lenti et al. (2022)
TGFβ	TGFβ controls Tregs thus reducing the development of autoimmune gastritis in a murine model, on the other hand controls fibrosis	To be define	To be define	—	Stummvoll et al. (2008)
TSLP	In AIG mice, TSLP negatively regulates the production of IL-12/23p40; TSLP is more expressed in AIG	Increased	Pejorative (To be validate)	Tezepelumab	Lenti et al. (2022)
eNAMPT	eNAMPT is increased in plasma and in organ culture biopsies of AIG. NAMPT increases IFNγ and IL-6 mucosal transcript	Increased	Pejorative	NCT05938036, ALT-100 (Not approved; phase II)	Lenti et al. (2022)

Notably, several biological therapies targeting these cytokines have already been approved or are in clinical trials for other inflammatory disorders (Table 1). For example, anti-TNF therapies (infliximab, adalimumab, etanercept, golimumab, and certolizumab pegol) are well-characterized for other gastrointestinal disorders. Infliximab is approved for conditions such as ulcerative colitis (UC), Crohn’s disease (CD), rheumatoid arthritis (in combination with methotrexate), psoriatic arthritis, and ankylosing spondylitis. On the other hand, tocilizumab, a neutralizing antibody against IL-6, is approved for treating active rheumatoid arthritis in patients who have had an inadequate response to one or more disease-modifying anti-rheumatic drugs. Tocilizumab is also in clinical trials for COVID-19 pneumonitis, lung transplantation, neuromyelitis optical spectrum disorder, and acute chest syndrome. Currently, also the anti-IFNγ is available, since emapalumab, an anti-IFNγ antibody, is approved for hemophagocytic lymphohistiocytosis and is being investigated in clinical trials for systemic juvenile idiopathic arthritis, SARS-CoV-2 infection, and aplastic anemia. A possible therapy could be targeting IL-13, thought tralokinumab, an anti-IL-13 antibody, that is approved for atopic dermatitis in patients inadequately controlled with topical therapies and is in trials for idiopathic pulmonary fibrosis and corticosteroid-dependent asthma.

Finally, a possible option could be the anti-IL-17 therapy, including secukinumab, ixekizumab, and brodalumab, which are now approved for severe hidradenitis suppurativa and plaque psoriasis. To target TSLP, tezepelumab, a human IgG2 monoclonal antibody that inhibits the binding of TSLP to its receptor, is approved in the European Union for treating severe asthma not controlled with high-dose corticosteroids. Its importance in other gastrointestinal disorders, such as eosinophilic esophagitis, is under investigation (clinicaltrial.gov). Regarding eNAMPT, as noted above and in Table 1, an eNAMPT-neutralizing mAb (ALT-100), has been developed and one is in phase II trials for ARDS (Travelli and Garcia, Aqualung) (NCT05938036), and should be considered as a potential therapeutic strategy in AIG. In summary, although there are no approved biological therapies for AIG yet, exploring these cytokine targets could lead to new treatment options. Further research is crucial to validate the contribution of these cytokines in AIG and to develop effective therapeutic strategies.

## 4 Conclusion and future perspectives

Autoimmune gastritis (AIG) is an autoimmune disorder characterized by increased inflammation which predisposes patients that are affected to the development of gastric adenocarcinoma and type I neuroendocrine tumors. The exact pathogenesis of this autoimmune disorder is still under investigation, and the dysregulation of the immunological mechanisms in autoimmune gastritis are essential for the discovery of potential pharmacological therapies. In this review, we have discussed the current knowledge on pro and anti-inflammatory cytokines in autoimmune gastritis, shedding light on possible relevant cytokines that may be further studied in the next future in both murine models and translational studies to define putative biological therapies for patients affected of AIG.
